# The beneficial effect of sulforaphane on platelet responsiveness during caloric load: a single-intake, double-blind, placebo-controlled, crossover trial in healthy participants

**DOI:** 10.3389/fnut.2023.1204561

**Published:** 2023-07-06

**Authors:** Hidde P. van Steenwijk, Evi Winter, Edward Knaven, Jos F. Brouwers, Myrthe van Baardwijk, Jasper B. van Dalum, Teus J. C. Luijendijk, Frits H. M. van Osch, Freddy J. Troost, Aalt Bast, Khrystyna O. Semen, Alie de Boer

**Affiliations:** ^1^Food Claims Centre Venlo, Faculty of Science and Engineering, Maastricht University, Maastricht, Netherlands; ^2^Research Group Analysis Techniques in the Life Sciences, Avans University of Applied Sciences, Breda, Netherlands; ^3^Omnigen B.V., Delft, Netherlands; ^4^Department of Pathology and Clinical Bioinformatics, Erasmus MC, University Medical Center Rotterdam, Rotterdam, Netherlands; ^5^Stichting Control in Food and Flowers, Delfgauw, Netherlands; ^6^Department of Clinical Epidemiology, VieCuri Medical Center, Venlo, Netherlands; ^7^Department of Epidemiology, NUTRIM, Faculty of Health, Medicine and Life Sciences, Maastricht University, Maastricht, Netherlands; ^8^Division of Gastroenterology-Hepatology, Department of Internal Medicine, School of Nutrition and Translational Research in Metabolism, Maastricht University Medical Center+, Maastricht, Netherlands; ^9^Food Innovation and Health, Centre for Healthy Eating and Food Innovation, Maastricht University, Maastricht, Netherlands; ^10^University College Venlo, Faculty of Science and Engineering, Maastricht University, Maastricht, Netherlands; ^11^Department of Pharmacology and Toxicology, Faculty of Health, Medicine and Life Sciences, Maastricht University, Maastricht, Netherlands

**Keywords:** immunothrombosis, inflammation, dietary antiplatelets, phytonutrients, thromboxanes, nutrition, non-communicable diseases

## Abstract

**Background and aims:**

As our understanding of platelet activation in response to infections and/or inflammatory conditions is growing, it is becoming clearer that safe, yet efficacious, platelet-targeted phytochemicals could improve public health beyond the field of cardiovascular diseases. The phytonutrient sulforaphane shows promise for clinical use due to its effect on inflammatory pathways, favorable pharmacokinetic profile, and high bioavailability. The potential of sulforaphane to improve platelet functionality in impaired metabolic processes has however hardly been studied in humans. This study investigated the effects of broccoli sprout consumption, as a source of sulforaphane, on urinary 11-dehydro-thromboxane B_2_ (TXB_2_), a stable thromboxane metabolite used to monitor eicosanoid biosynthesis and response to antithrombotic therapy, in healthy participants exposed to caloric overload.

**Methods:**

In this double-blind, placebo-controlled, crossover trial 12 healthy participants were administered 16g of broccoli sprouts, or pea sprouts (placebo) followed by the standardized high-caloric drink PhenFlex given to challenge healthy homeostasis. Urine samples were collected during the study visits and analyzed for 11-dehydro-TXB_2_, sulforaphane and its metabolites. Genotyping was performed using Illumina GSA v3.0 DTCBooster.

**Results:**

Administration of broccoli sprouts before the caloric load reduced urinary 11-dehydro-TXB_2_ levels by 50% (*p* = 0.018). The amount of sulforaphane excreted in the urine during the study visits correlated negatively with 11-dehydro-TXB_2_ (*r_s_* = −0.377, *p* = 0.025). Participants carrying the polymorphic variant NAD(P)H dehydrogenase quinone 1 (NQO1*2) showed decreased excretion of sulforaphane (*p* = 0.035).

**Conclusion:**

Sulforaphane was shown to be effective in targeting platelet responsiveness after a single intake. Our results indicate an inverse causal relationship between sulforaphane and 11-dehydro-TXB_2_, which is unaffected by the concomitant intake of the metabolic challenge. 11-Dehydro-TXB_2_ shows promise as a non-invasive, sensitive, and suitable biomarker to investigate the effects of phytonutrients on platelet aggregation within hours.

**Clinical trial registration:**

[https://clinicaltrials.gov/], identifier [NCT05146804].

## 1. Introduction

Our understanding of platelet functionality has changed dramatically over the past decade. Platelets are no longer viewed simply as hemostasis regulators; they are now recognized as crucial in coordinating inflammatory and immune responses (1, 2). Impaired platelet function has been observed in several chronic inflammatory conditions, including thrombosis and thrombotic disorders (3), asthma (4), myocardial infarction (5), unstable angina pectoris (6), atherosclerosis (7), and type 2 diabetes (8, 9). Interestingly, also postprandial hyperglycemia has been shown to cause platelet activation (10) and promote inflammatory state in healthy individuals (11–13). In the clinical setting, administering caloric loads to humans has been used as a tool to pressurize adaptive mechanisms and trigger inflammation which may also be associated with an increase in platelet reactivity (14).

Thromboxanes are arachidonic acid metabolites with significant biological activity, including regulation of platelet functionality (15–17). Increased production of thromboxanes was recognized to contribute to vasculopathy by adversely affecting endothelial function and promoting vascular inflammation (18). By activating the thromboxane receptor, those metabolites cause significant alterations of platelet shape, inside-out activation of integrins, and degranulation (15–17), which might subsequently lead to increased platelet aggregation and thrombosis. On the other hand, increased production of thromboxane A and activation of platelets are recognized to be crucial modulators of the functionality of multiple inflammatory pathways involved in the progression of the cardiovascular diseases (19). 11-Dehydro-thromboxane B_2_ (TXB_2_) is produced from the breakdown of thromboxane A and can be measured in urine as a relevant marker of platelet reactivity, e.g., to monitor the response to aspirin (ASA) therapy when used to prevent heart disease (20, 21).

In fact, targeting thromboxane production via antiplatelet agents has become a cornerstone of cardiovascular disease treatment (1, 22). Antiplatelet drugs (i.e., ASA, clopidogrel, prasugrel and abciximab) typically irreversibly suppress a specific pathway of platelet aggregation (1, 22). Therefore, the main limitations of current antiplatelet agents include the risk of bleeding and prolonged duration of action that cannot be reversed if the need for hemostasis or emergency surgery arises (22, 23), which limits their use in many clinical situations, e.g., prophylaxis of cardiovascular events in high-risk groups (1). Phytonutrients have also been shown to affect platelet functionality with water-soluble tomato concentrate (WSTC) being the first to be recognized by the European Food Safety Agency (EFSA) as functional food that helps to maintain normal platelet aggregation, which contributes to healthy blood flow (24). Unlike drug therapy, the antiplatelet effect of most phytochemicals is reversible, making them potentially safe for use in a variety of clinical scenarios, including primary prevention of cardiovascular diseases (17.9 million deaths annually) in the general population (1, 25, 26). Therefore, it is paramount to search for novel nutritional strategies that support platelet functionality.

Of the various plant-derived bioactive nutrients, sulforaphane holds the most promise for clinical testing due to superior absorption and pharmacokinetic profile (27–46). Glucoraphanin, the biogenic precursor of sulforaphane, is present in large amounts in broccoli and to a lesser extent in other Brassica species (47, 48). Since glucoraphanin is biologically inert, factors controlling the biosynthesis of glucoraphanin and conversion to sulforaphane by the enzyme myrosinase are of great importance to the potential health effects of these vegetables (28, 39, 48). The antithrombotic properties of sulforaphane have been demonstrated in animals and *in vitro* studies (49, 50). Sulforaphane exerts antiplatelet activity which may initially activate adenylate cyclase/cAMP, followed by reversible inhibition of multiple intracellular signals such as the PI3-kinase/Akt and PLCγ2-PKC-p47 cascades (49, 50). Although promising, demonstrating an antithrombotic effect *in vivo* after oral administration of the compound has been scarcely explored in clinical trials, hence sulforaphane’s ability to modulate platelet functionality in humans is still poorly studied (51).

These findings motivated us to investigate the effects of broccoli sprout consumption, as a source of sulforaphane, on urinary 11-dehydro-TXB_2_ in healthy participants exposed to a standardized caloric load and to investigate interindividual genetic variability.

## 2. Methods

A randomized, placebo-controlled, double-blind study was conducted with a cross-over design. The study protocol (NL77272.068.21) was approved by the Medical Ethics Review Committee of Maastricht University Medical Centre+ (MUMC+) and Maastricht University, Maastricht, the Netherlands, and performed in full accordance with the declaration of Helsinki of 1975 as revised in 2013, Fortaleza, Brazil (52). The trial registration number within ClinicalTrials.gov is NCT05146804. All subjects provided written informed consent to participate.

### 2.1. Subjects

Healthy men and women were recruited by local and social media advertisements. Inclusion criteria were that participants were between 18 and 50 years old, had a body mass index (BMI) between 18.5 and 30 kg/m^2^, with a stable weight (<5% body weight change) and constant eating habits over the past 3 months. Exclusion criteria were the previous diagnosis of an inflammatory condition or disease or a history of hypothyroidism, chronic kidney or/and liver disorders, coronary artery disease, malignant hypertension, seizures, involved in intensive sports activities more than four times a week or at top sport level, regular intake of medication that may affect inflammatory response including NSAIDs, psychotic, addictive, or other mental disorders, aversion, intolerance or allergy to cruciferous vegetables and/or palm olein, dextrose, protein supplement, vanilla aroma, the use of dietary supplements with potential effects on antioxidant or inflammatory status and/or viral or bacterial infections requiring the use of antibiotics, laxatives and anti-diarrheal drugs 4 weeks prior to inclusion, excessive alcohol consumption (≥28 consumptions, approx. 250 g alcohol per week), pregnancy and/or breastfeeding, reported slimming or medically prescribed diet, as well as adhering to a vegetarian or vegan lifestyle.

### 2.2. Study design and procedures

**Figure 1:**
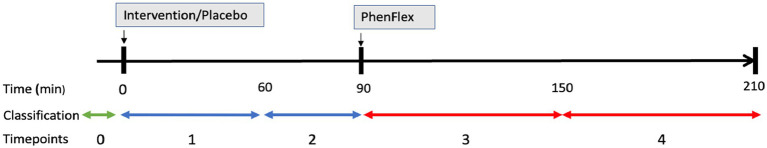
Schematic presentation of a study visit. Administration of intervention (sulforaphane/placebo) was followed in 90 min by administration of standardized caloric challenge PhenFlex. Urine samples were classified into three groups: **(A)** (baseline, green line), **(B)** (after intervention or placebo, blue lines), and **(C)** (after PhenFlex challenge, red lines). In addition, samples were also divided into 5 timepoints: 0 (baseline), 1 (<60 min after intervention or placebo), 2 (>60 min after intervention or placebo), 3 (<60 min after PhenFlex challenge), and 4 (>60 min after PhenFlex challenge).

Commercially available broccoli sprouts BroccoCress^®^, a rich source of sulforaphane, were used as the experimental product. In total, 16 g of sprouts were used per serving. Sulforaphane (BroccoCress^®^) and placebo (Affilla Cress^®^) were administered to each participant in the randomized fashion on different testing days. The period between two visits was 7 ± 3 days. Information about demographics, alcohol consumption, and anthropometric data were assessed on the first visit. BMI, total body fat and visceral fat were measured using the Omron BF511R^®^ monitor. The same testing scheme was applied during two visits (Figure 1), i.e., each participant received a single serving of intervention/placebo, which after 90 min was followed by oral administration of the PhenFlex challenge. Urine samples were collected throughout the day of the visit, preferably before the intervention/placebo, between intervention/placebo and the PhenFlex challenge, and after the PhenFlex administration. All participants were instructed to come fasted to each visit, to avoid consumption of broccoli or other cruciferous vegetables 2 days before each visit and to refrain from intense physical activity on the day of the visit. During the visit, participants remained in the testing location and were allowed to drink water *ad libitum*. No food intake was permitted during the visit.

### 2.3. Intervention and caloric challenge (PhenFlex)

Shortly (maximum of 3 min) before administration, the sprouts were cut approximately 1 cm below the leaves, weighed, and mashed with a small amount of tap water (approximately 13°C) in a kitchen blender for 30s at room temperature (Premium Impuls Blender Smoothiemaker; Impuls; 180 W). Subsequently, tap water (approximately 13°C) was added to a total amount of 250 mL and participants were instructed to drink the entire mixture. Commercially available pea sprouts (Affilla Cress^®^) were used as placebo in this study since pea sprouts do not contain glucoraphanin/sulforaphane. Affilla Cress (16 g) was prepared and administered in a similar fashion. Blinding of participants was ensured by the even appearance of both drinks and the use of nasal plugs during consumption of the investigational products. The placebo or intervention product was prepared by a researcher who was not involved in any other study procedures and data analysis. Ninety minutes after administration of the investigational products, participants were asked to drink a high-fat, high-glucose, high-caloric product (PhenFlex) (53). For the preparation of the PhenFlex (400 mL, 950 kcal) 60 g palm olein, 75 g dextrose, 20 g protein, 0.5 g artificial vanilla aroma and 320 mL tap water were used (53). In all cases, PhenFlex mixtures were freshly prepared, and the participants were instructed to consume the drink within 5 min.

### 2.4. Urine sampling and assessment of 11-dehydro-TXB_2_

Urine samples were collected throughout the days of the visits in pre-labeled containers. The total volume and time of collection of each sample was recorded. Samples were aliquoted and stored at ≤ −80°C until the day of analysis. Urine samples were analyzed for 11-dehydro-TXB_2_ using an enzyme-linked immunoassay kit (UTxB2: assay #519510, Cayman Chemical, Ann Arbor, MI) following the manufacturer’s instructions. The manufacturer recommended standardizing urinary 11-Dehydro-TXB_2_ values for creatinine levels using a colorimetric assay kit [Creatinine (urinary) Colorimetric Assay Kit: assay #500701, Cayman Chemical, Ann Arbor, MI]. 11-dehydro-TXB_2_ concentrations were normalized to urinary creatinine concentrations expressed as μg/mg Cr to account for inter-individual variability in urine dilution. Urine samples were classified into three groups for analysis: A (baseline), B (after intervention or placebo) and C (after PhenFlex challenge). In addition, samples were also divided into five timepoints: 0 (baseline), 1 (<60 min after intervention or placebo), 2 (>60 min after intervention or placebo), 3 (<60 min after PhenFlex challenge) and 4 (>60 min after PhenFlex challenge).

### 2.5. Measurement of urinary sulforaphane and metabolites

The determination of sulforaphane (SFN) and sulforaphane-glutathione (SFN-GSH), sulforaphane-cysteine (SFN-Cys), sulforaphane-cysteine-glycine (SFN-CG) and sulforaphane-N-acetylcysteine (SFN-NAC) in human urine was based on an HPLC–MS/MS method from Egner et al. (54).

### 2.6. Materials

Trc Canada deuterated stable Isotope solutions of sulforaphane-d8 and sulforaphane-d8-N-acetyl-L-cysteine were purchased from LGC standards (Wesel, Germany). J.T. Baker ethanol and Biosolve acetonitrile were purchased from Boom (Meppel, the Netherlands). Formic acid (FA) was purchased from VWR (Amsterdam, the Netherlands). Fresh water was obtained from an inhouse MilliQ Advantage A10 system containing a LC–MS filter pack (Millipore, Burlington, MA). All chemicals were of analytical quality or higher.

#### 2.6.1. Internal standard preparation

The stock solutions of SFN-d8-NAC and SFN-d8 were diluted with ethanol containing 0.2% FA by a factor of 10 and 100, respectively. From both dilutions 40 μL was taken and combined in a new tube. The mixture was further diluted with ethanol containing 0.2% FA to give final concentrations of 2 μg/mL for SFN-d8-NAC and 0.2 μg/mL for SFN-d8 (IS mix). Stability of IS mix was verified by repeated analysis over a time period of several days.

#### 2.6.2. Sample preparation

Urine samples were taken from the −80°C storage and thawed on ice. The samples were vortexed briefly and centrifuged at 21000 *g* and 4°C for 5 min. From the supernatant 20 μL was transferred to a HPLC vial with a 200 μL insert. IS mix (20 μL) was added and mixed with the urine sample. Thereafter, the samples (5 μL) were directly injected into the analytical system.

#### 2.6.3. Instrumental and data processing

For chromatographic separation an ExionLC UHPLC system (AB Sciex Framingham, MA) equipped with a binary pump, a thermostated autosampler and a column oven was used to maintain a constant temperature (40°C) during the analytical run. For detection a X500R qToF MS system (AB Sciex Framingham, MA) was used. Data acquisition was performed using SciexOS V2.1.6.59781 (AB Sciex). Data processing and identification of sulforaphane and its metabolites was performed by MS-DIAL (version 4.92).

### 2.7. Genotyping

The participants were requested to take a DNA sample using cheek swabs. Subsequently, all samples were processed at the Human Genomics Facility (HuGe-F) of the Genetic Laboratory of the Department of Internal Medicine at Erasmus MC using the Illumina GSA v3.0 DTC array consisting of 703,320 unique Single Nucleotide Polymorphisms (SNPs). The HuGe-F applied genotyping using the GenomeStudio v2 software with in-house cluster files for reference genome GRCh37. The resulting genotype files are subjected to in-house quality control pipelines which includes correcting for missing data and removing SNPs violating Hardy–Weinberg Equilibrium (55). Finally, imputation is applied to the corrected and filtered SNPs with Beagle software (56) using a reference panel from the 1.000 Genomes project consisting of 2,141 samples (57). Seven gene regions were selected for the analysis based on literature review (36, 58–64). For these genes, their genomic location according to the GRCh37 reference genome was determined from the NCBI Gene database and can be viewed in Table 1 (72). The preprocessed SNPs were filtered on overlap with these gene regions. The resulting subset of potentially interesting SNPs was annotated with information from the NCBI dbSNP database (72).

**Table 1:** Selected genes based on sulforaphane (SFN) metabolism and effects.

Gene name	Chromosome	Start position (bp)	End position (bp)	Influence on sulforaphane metabolism and/or effects of sulforaphane in humans
GSTM1	1	110,230,439	110,236,367	GSTM1-null mutations may benefit more from SFN, due to decreased metabolism (58, 59, 65).
GSTP1	11	67,351,283	67,354,124	GSTP1Ile105Val genotypes may benefit more from SFN, due to decreased metabolism (66, 67).
GSTT1	22	24,376,133	24,384,311	GSTT1-null mutations may benefit more from SFN, due to decreased metabolism (58, 59, 65).
NQO1	16	69,743,304	69,760,463	NQO1 polymorphisms may indirectly affect SFN metabolism (58).
CYP1A2	15	75,041,186	75,048,948	CYP1A2 polymorphisms may influence the effects of SFN (59).
UGT1A1	2	234,668,916	234,681,946	UGT1A1 polymorphisms may influence the effects of SFN (62, 68–70).
NAT2	8	18,248,792	18,258,728	NAT2 polymorphisms may influence the effects of SFN (36, 71).

The genomic locations are listed according to the GRCh37 reference genome and the NCBI gene database.

### 2.8. Statistical analysis

All normally distributed data are presented as mean ± standard deviation (SD). The non-normally distributed data are shown as median (interquartile range). For categorical variables, frequency and/or percentages are presented. Differences between the groups were assessed by repeated measures ANOVA for normally distributed parameters, or Skillings-Mack tests for the data that was not normally distributed. In addition, paired sample *t*-tests and Wilcoxon rank-sum tests were performed as *post-hoc* tests for the normally distributed samples and the non-normally distributed samples, respectively. Mann–Whitney *U* tests were performed to compare the change in urinary 11-dehydro-TXB_2_ within sample groups between subjects. To study the association between 11-dehydro-TXB_2_ and sulforaphane (metabolites), Spearman correlation was performed. The tool Stargazer (73) was used for phenotyping the metabolizer type of the participants for the NAT1 and NAT2 pharmacogenes. Participants were subdivided into low versus high excretion of sulforaphane. An adaptation of Fisher’s Exact test within the plink genomics software was applied on the groups to test for significantly different genotypes (74). Additionally, adaptive permutation testing within plink was executed to test the validity of the resulting *p*-values. All analyses were performed two-tailed with *p* ≤ 0.05 considered statistically significant.

## 3. Results

### 3.1. Subject characteristics

**Figure 2:**
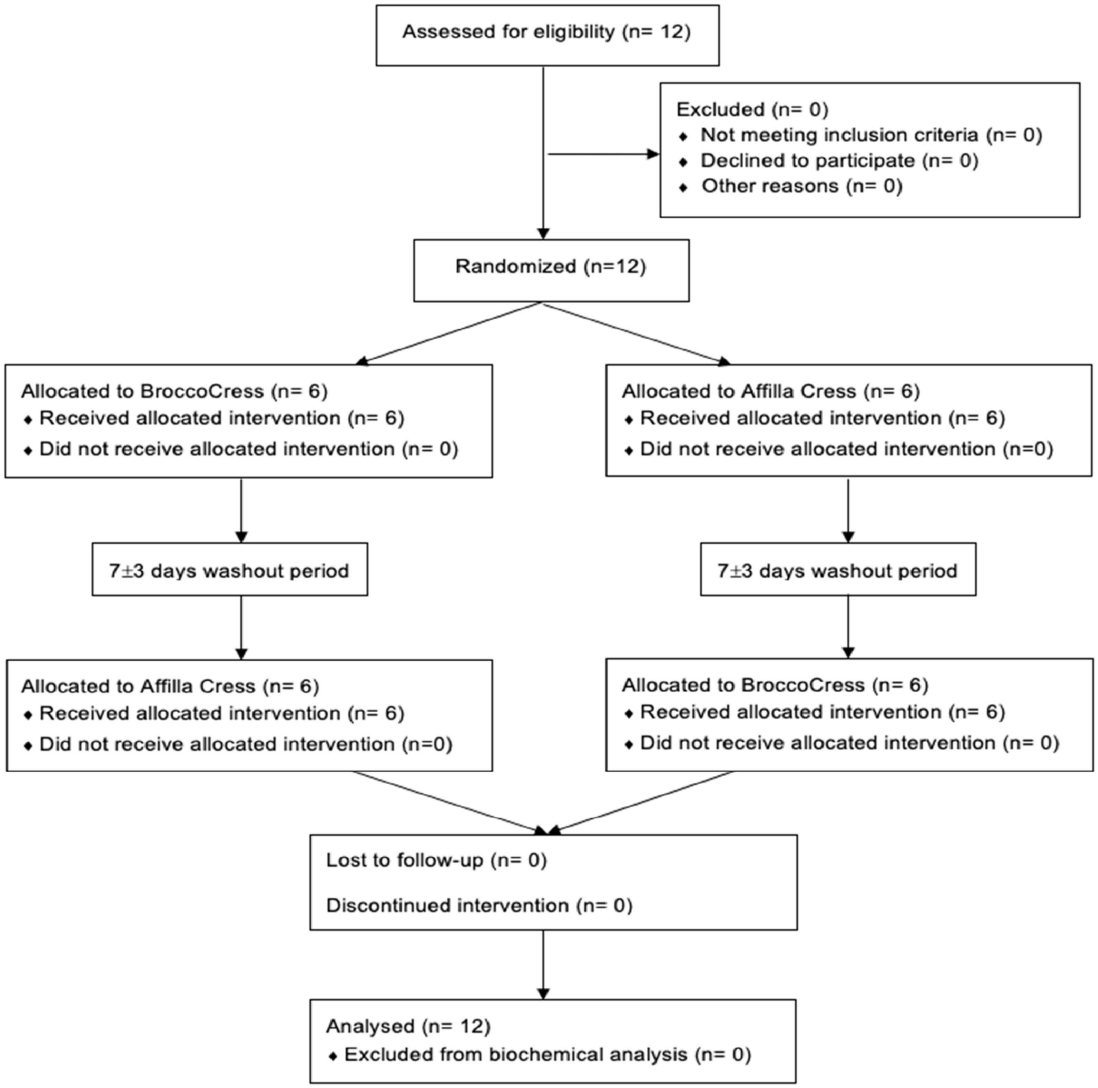
CONSORT flow diagram.

Between November 2021 and January 2022, a total of 12 subjects were found to be eligible to participate in the present study and were randomly allocated to either initial administration of sulforaphane or placebo. Baseline characteristics of the study population (*n* = 12) are summarized in Table 2. All participants completed the study and were included in the data analysis for biochemical testing (Figure 2). A total of 54 urine samples were collected and assessed. Urine samples were classified into three groups: A (baseline), B (after intervention or placebo) and C (after PhenFlex challenge). In addition, samples were also divided into five timepoints: 0 (baseline), 1 (<60 min after intervention or placebo), 2 (>60 min after intervention or placebo), 3 (<60 min after PhenFlex challenge) and 4 (>60 min after PhenFlex challenge). Given the crossover design of the study, a pre-test was first performed to verify whether the time of the washout period was sufficient and to exclude any carry-over effects. The pre-test (Wilcoxon rank sum test) revealed no differences between treatment allocations for 11-dehydro-TXB_2_ (*z* < −4.47, *p* > 0.655). A significant difference between the time of sampling in each sample group was checked by Kruskal-Wallis test H (5) = 63.647, *p* < 0.001.

**Table 2:** Characteristics of the study participants [mean (SD)].

Characteristics	Population (*n* = 12)
Sex (n, %)	
Female	1 (8.3)
Male	11 (91.7)
Age (years)	26.9 (3.6)
BMI (kg/m^2^)	23.1 (1.6)
Body Fat (%)	
Female	28.9 (n/a)
Male	21.4 (3.1)
All	22.0 (3.6)
Visceral fat level	5.17 (1.57)
Alcohol consumption, n (%)	
Moderate	0 (0)
Heavy	9 (75)
Very heavy	3 (25)
Smoking status, n (%)	
Smoker	5 (42)
Non-smoker	7 (58)

### 3.2. The effect of sulforaphane on 11-dehydro-TXB_2_

**Figure 3:**
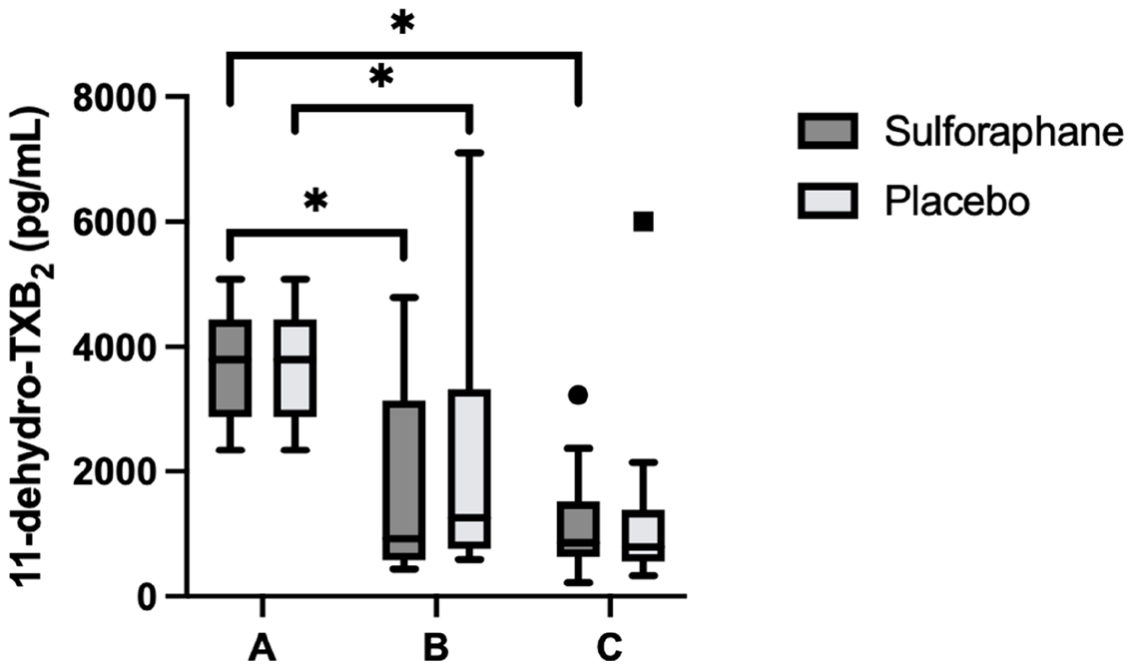
Changes in urinary 11-dehydro-TXB_2_ concentration in sulforaphane and placebo groups. Uncorrected data are presented as boxplots [median, interquartile range, outliers (circles) and far outliers (squares)]. Urine samples were clustered into three timepoints: **(A)** (baseline), **(B)** (after intervention or placebo), and **(C)** (after PhenFlex challenge). *p < 0.05.

**Figure 4:**
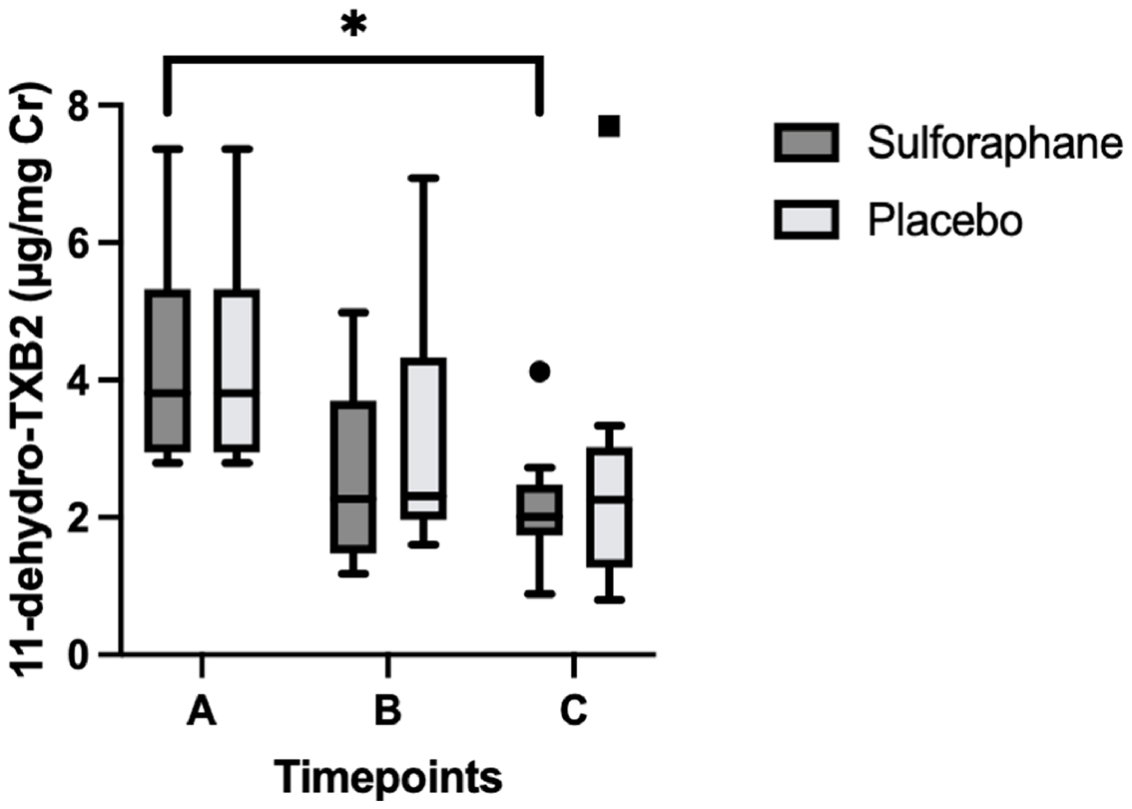
Changes in urinary 11-dehydro-TXB_2_ concentration in sulforaphane and placebo groups corrected for creatinine. Data are presented as boxplots [median, interquartile range, outliers (circles) and far outliers (squares)]. Urine samples were clustered into three timepoints: **(A)** (baseline), **(B)** (after intervention or placebo), and **(C)** (after PhenFlex challenge). *p < 0.05.

Differences between groups were assessed for uncorrected and creatinine-corrected 11-dehydro-TXB_2_. For the uncorrected data, a significant difference was observed in urinary 11-dehydro-TXB_2_ concentrations between all groups in the sulforaphane group (X^2^(2) = 11.60, *p* = 0.003) and in the placebo group (X^2^(2) = 7.58, *p* = 0.023). *Post hoc* analysis showed significant differences for the sulforaphane group between samples A and B, and between samples A and C (*p* = 0.028 and *p* = 0.018), and between samples A and B for placebo (*p* = 0.028; Figure 3). Analysis of the corrected data showed a significant difference in urinary 11-dehydro-TXB_2_ concentration between all groups in the sulforaphane group (X^2^(2) = 9.27, *p* = 0.010), no significant differences were observed in the placebo group (X^2^(2) = 3.53, *p* = 0.171). *Post hoc* analysis showed a significant difference in the sulforaphane group between samples A and C for corrected data (*p* = 0.018; Figure 4). Analysis between treatment allocations within samples showed no significant results (Table 3).

**Table 3:** Uncorrected and corrected urinary 11-dehydro-TXB_2_ concentrations (mean ± SD) per timepoint per treatment allocation.

		Population (*n* = 12)				
Treatment	Timepoints^^^	0 (A)	1 (B)	2 (B)	3 (C)	4 (C)
Sulforaphane	Samples (n)	7	6	5	10	7
	Time of sampling (median (IQR))	n/a	45 (28)	75 (43)	120 (38)	180 (40)
	Sample volumes [median (IQR)]	90 (93)	383 (485)	240 (330)	418 (380)	380 (490)
	Uncorrected TXB_2_ (pg/mL)	3671 ± 860	4115 ± 7247	2076 ± 1637	1131 ± 1132	1106 ± 843
	Corrected TXB_2_ (μg/mg Cr)	4.21 ± 1.51	3.74 ± 4.38	3.21 ± 1.08	2.07 ± 1.07	2.12 ± 1.02
Placebo	Samples (n)	7	7	7	6	11
	Time of sampling [median (IQR)]	n/a	45 (20)	85 (30)	118 (16)	200 (35)
	Sample volumes [median (IQR)]	90 (93)	240 (320)	260 (310)	400 (278)	350 (150)
	Uncorrected TXB_2_ (pg/mL)	3671 ± 860	2908 ± 2927	1839 ± 1540	618 ± 154	1357 ± 1558
	Corrected TXB_2_ (μg/mg Cr)	4.21 ± 1.51	3.74 ± 2.44	2.78 ± 1.26	1.48 ± 0.55	2.67 ± 1.85

Cr – corrected for creatinine (mg/mL). ^^^ Timepoints clustered in three groups: 0 = A (baseline), 1 + 2 = B (after intervention or placebo) and 3 + 4 = C (after PhenFlex challenge).

### 3.3. Sulforaphane excretion

**Figure 5:**
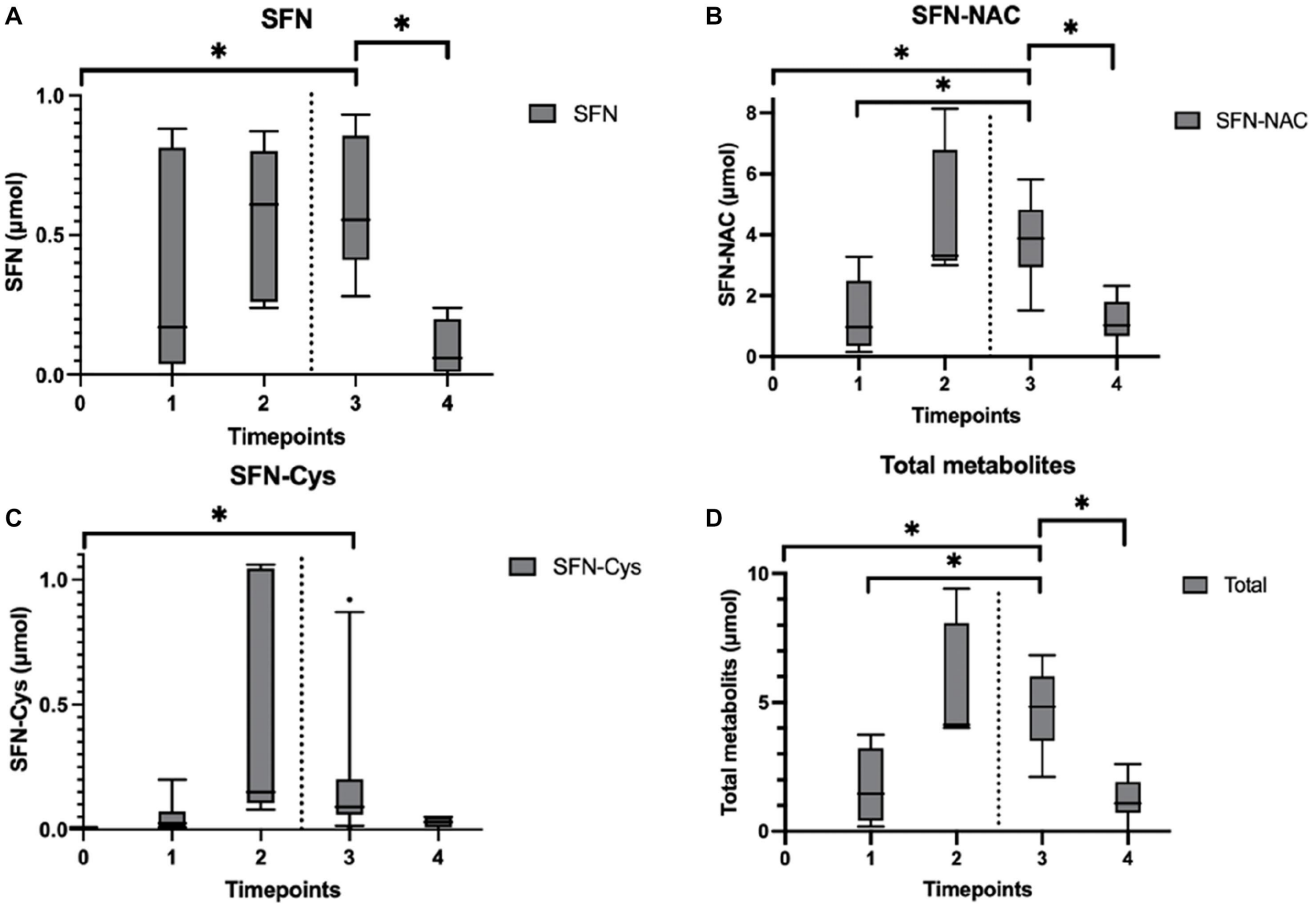
Urinary sulforaphane (SFN), sulforaphane-cysteine (SFN-Cys), sulforaphane-N-acetylcysteine (SFN-NAC), and total metabolites excretion after participants consumed a single intake (16 g) of broccoli sprouts. Data are presented as boxplots [median, interquartile range, and outliers (circles)]. The dashed lines indicate when the PhenFlex challenge was administered. Urine samples were clustered into five timepoints: 0 (baseline), 1 (<60 min after intervention), 2 (>60 min after intervention), 3 (<60 min after PhenFlex challenge), and 4 (>60 min after PhenFlex challenge). The amount of SFN **(A)** excreted in the urine correlated negatively with 11-dehydro-TXB_2_ (*r_s_* = −0.377, *p* = 0.025). Moderate strength, inverse correlations between other metabolites were observed, i.e., SFN-NAC **(B)** (*r_s_* = −0.210, *p* = 0.225), SFN-Cys **(C)** (*r_s_* = −0.131, *p* = 0.452) and total metabolites **(D)** (*r_s_* = −0.196, *p* = 0.258). *p < 0.05.

The presence of sulforaphane after consuming fresh broccoli sprouts, and absence after placebo, was determined in urine samples collected during the study visits, indicating an adequate period of abstinence (mean total amount of sulforaphane and metabolites: 8.2 vs. 0.4 μmol, *p* < 0.001). Sulforaphane, SFN-N-acetylcysteine (SFN-NAC), SFN-cysteine, and total amount of metabolites were determined in all urine samples. No detectable levels of SFN-glutathione and SFN-cysteine-glycine were quantified. In the intervention group, the amount of sulforaphane excreted in urine significantly increased 120 min after its administration (*p* = 0.028) and decreased during the subsequent hour (*p* = 0.043; Figure 5A). The amount of SFN-NAC excreted in urine significantly increased 120 min after the intervention (*p* = 0.028), and from the first 45 min to 1.5 h afterwards (*p* = 0.028) decreased during the subsequent hour (*p* = 0.043; Figure 5B). The amount of SFN-cysteine excreted in urine significantly increased 2 h after the intervention (*p* = 0.028; Figure 5C). The total amount of metabolites excreted in urine significantly increased 120 min after the intervention (*p* = 0.028) and from the first 45 min to an hour and a half afterwards (*p* = 0.046) and decreased during the subsequent hour (*p* = 0.043) (Figure 5D). In addition, the amount of sulforaphane excreted in the urine during the study visits correlated negatively with 11-dehydro-TXB_2_ (*r_s_* = −0.377, *p* = 0.025). Moderate strength, inverse correlations between other metabolites SFN-NAC (*r_s_* = −0.210, *p* = 0.225), SFN-Cys (*r_s_* = −0.131, *p* = 0.452) and total metabolites (*r_s_* = −0.196, *p* = 0.258) were observed.

### 3.4. The influence of single nucleotide polymorphisms on sulforaphane and 11-dehydro-TXB_2_

The 703,320 SNPs were subjected to the quality control pipeline, after which 624,240 SNPs were left. The subsequent imputation with Beagle resulted in 24,232,949 SNPs available for downstream analysis. Seven genes were selected for further analysis based on previous literature. Filtering the imputed SNPs using the gene regions as defined in Table 1 resulted in 77 SNPs that were further annotated and tested. Based on dbSNP annotations these included seven missense, 63 intronic, three synonymous, three 3′ untranslated regions (UTR), and one 5′ prime UTR variants. Testing the 77 relevant SNPs using Plink’s adoption of Fisher’s Exact test resulted in six SNPs associated with sulforaphane excretion (Table 4). All SNPs were found in the NQO1 gene and all, but one were intronic variants, while the other SNP was a missense variant causing a change from proline to serine. Participants carrying this polymorphic variant NQO1*2, which is characterized by a C609T (rs1800566, Pro187Ser) polymorphism of the NQO1 gene, showed decreased excretion of sulforaphane during the study visits [6.2 (1.0) vs. 9.2 (1.6) μmol total metabolites, *p* = 0.035]. No influence of these SNPs on 11-dehydro-TXB_2_ levels was observed. No influence of SNPs in GSTM1, GSTP1, GSTT1, CYP1A2, UGT1A1 and NAT2 genes, or type of NAT1 and NAT2 metabolizer on sulforaphane excretion and TXB_2_ levels was observed (Supplementary Tables 1, 2).

**Table 4:** Single nucleotide polymorphisms (SNPs) associated with sulforaphane excretion.

Gene name	Reference SNP ID (RsID)	Chromosome	Base pair (bp) position	Type	Consequence (missense only)	Fisher’s exact test *p*-value	Permutation *p*-value
NQO1	rs1800566	16	69,745,145	Missense	Proline→Serine	0.087	0.035
NQO1	rs57964521	16	69,750,092	Intron	–	0.087	0.035
NQO1	rs7186002	16	69,751,065	Intron	–	0.087	0.035
NQO1	rs1437135	16	69,757,828	Intron	–	0.087	0.035
NQO1	rs2196574	16	69,758,076	Intron	–	0.087	0.035
NQO1	rs2361839	16	69,758,395	Intron	–	0.087	0.035

The genomic locations are listed according to the GRCh37 reference genome and the NCBI gene database.

## 4. Discussion

The aim of this work was to investigate the effects of sulforaphane on the arachidonic acid metabolite, as well as the relationship between 11-dehydro-TXB_2_ and sulforaphane and its mercapturic conjugates in urine. Therefore, we assessed the effect of a single intake of fresh broccoli sprouts on urinary 11-dehydro-TXB_2_ in healthy participants exposed to a standardized caloric load.

### 4.1. Sulforaphane decreased urinary 11-dehydro-TXB_2_ in young healthy subjects exposed to caloric overload

This study showed that consumption of 16 g of broccoli sprouts before a caloric overload reduced the amount of urinary 11-dehydro-TXB_2_ in healthy participants by 50%. These results are consistent with previous research examining the effects of sulforaphane on urinary biomarkers associated with inflammation. Medina et al. (51) showed that higher doses (30 g and 60 g) of broccoli sprouts significantly reduced urinary 11-dehydro-TXB_2_ levels by 91 and 94%, respectively, within 12 h in healthy subjects. The higher reduction found by Medina et al. (51) is probably due to the combination of the higher amount of broccoli sprouts given, the lack of a caloric challenge, and the longer time of observation. These results, additionally, indicate that urinary 11-dehydro-TXB_2_ shows promise as a sensitive and suitable biomarker to investigate the effects of phytonutrients on platelet aggregation, at least in young healthy participants who are metabolically challenged in a short period of time. For example, in this study, the amount of urinary inflammatory biomarker high-sensitivity C-reactive protein (hs-CRP) remained unchanged during the study visits and was only detectable in 33/54 samples (data not shown).

### 4.2. The amount of sulforaphane and 11-dehydro TXB_2_ excreted in the urine showed an inverse relationship during the caloric challenge

Our data on sulforaphane excretion and its relationship to urinary thromboxane metabolites shows a significant inverse correlation between sulforaphane and urinary 11-dehydro-TXB_2_ (*r_s_* = −0.377, *p* = 0.025) during the metabolic challenge. In contrast to previous studies, this inverse relationship was only significant for sulforaphane and not for other metabolites nor total metabolites, which may have been influenced by the co-ingestion of the caloric load and the shorter duration of urine collection (51). It is striking however, that from these results it can be concluded that the metabolic state apparently has no effect on the effect of sulforaphane on 11-dehydro-TXB_2_ levels.

Previous research has shown that a single administration of a standardized caloric load, the PhenFlex challenge, impacts relevant metabolic processes involved in maintaining or regaining homeostasis of metabolic health in healthy volunteers (12). Contrary to our expectations, the caloric overload did not increase urinary 11-dehydro-TXB_2_ measured 2 h after administration of placebo (pea sprouts). Although pea sprouts have a similar nutrient profile to broccoli sprouts, except presence of sulforaphane, the increased amount of retinol and beta-carotene may have counteracted the expected post-challenge increase in 11-dehydro-TXB_2_. Retinol and beta-carotene are transformed to retinoic acid (RA) by retinaldehyde dehydrogenases via numerous conversions (75). RA is mediated by two nuclear receptor families, the retinoic acid receptors, and the retinoid X receptors (RXR) (76). These receptors interact with retinoic acid response elements on the promoter regions of target genes, resulting in the activation of nuclear transcription factors (77, 78). The main antithrombotic effect of retinol and beta-carotene is based on the interference of RXR with the NF-κB pathway (79, 80). Inhibition of NF-κB disrupts platelet function by reducing its thrombogenic potential and shows promise when compounds that block NF-κB activation, such as sulforaphane and RA, are considered for prophylaxis of various thrombo-inflammatory diseases (80).

### 4.3. The NQO1*2 polymorphism may decrease sulforaphane excretion

Previous reports have shown that genetic predisposition may influence the bioavailability and excretion rate of sulforaphane, however results are incongruent (48, 58, 59, 61, 64, 81–87). Partially consistent with Boddupalli et al. (58), we found lower excretion of sulforaphane in participants carrying the polymorphic variant NQO1*2, but no effect of SNPs in other phase II detoxification enzyme genes.

**Figure 6:**
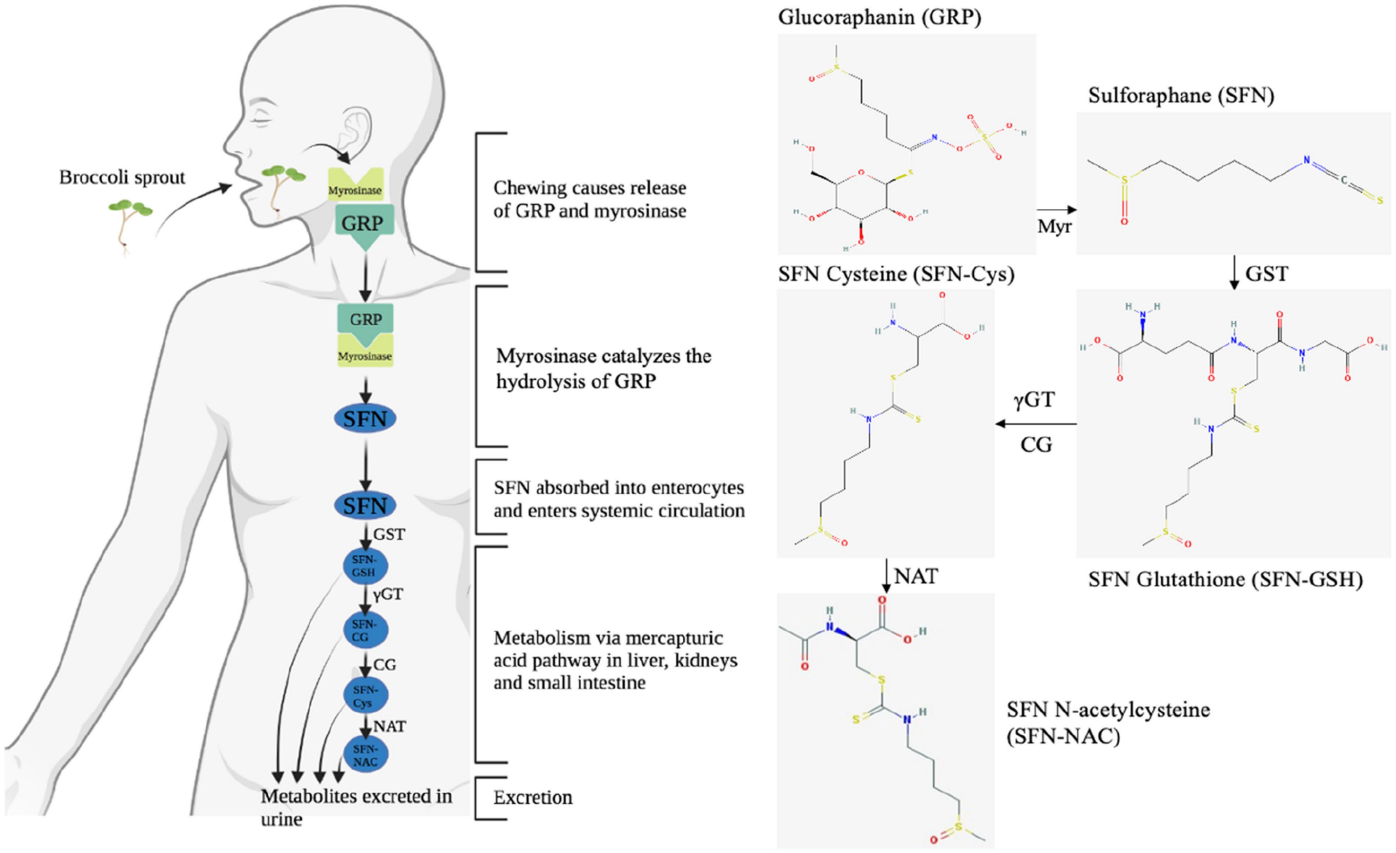
The enzymatic conversion of glucoraphanin (GRP) to sulforaphane (SFN) by myrosinase (Myr), followed by the absorption, distribution, metabolism, and excretion of SFN via the mercapturic acid pathway. The enzymatic conversion of GRP to SFN is considered to be the rate-limiting step affecting bioavailability within this cascade, as the efficiency of conversion of SFN to metabolites in humans is very high (70–90%) (48). GRP, glucoraphanin; GST, glutathione S-transferase; Myr, myrosinase; γGT, γ-glutamyl transferase; CG, cysteinylglycinase; NAT, N-acetyl transferase; SFN-Cys, sulforaphane-cysteine; SFN-NAC, sulforaphane-N-acetylcysteine; SFN-GSH, sulforaphane glutathione conjugate.

When cruciferous vegetables are damaged, such as when preparing or chewing, myrosinase is released. This enzyme is normally stored separately from glucosinolates, such as glucoraphanin, in different cells or in different intracellular compartments, depending on the plant species (44). Myrosinase catalyzes the hydrolysis of glucoraphanin to liberate the glucose group, resulting in an unstable aglucone that spontaneously rearranges to give rise to a range of products, of which sulforaphane is the most reactive (Figure 6) (44). Cooking cruciferous vegetables inactivates myrosinase, resulting in less sulforaphane formation (44). Bacterial populations of the gut microbiota are also able to converse glucoraphanin to sulforaphane; however, it is estimated to be approximately six-fold less effective than plant myrosinase (88). After this critical first step, sulforaphane is conjugated with glutathione (GSH) upon entry into the mammalian cell, catalyzed by glutathione S-transferases (GSTs), in the liver, entering the mercapturic acid pathway (89). The glutathione conjugate is subjected to a series of sequential conversions, resulting in the *N*-acetylcysteine conjugate (mercapturic acid) as the main metabolite, which is excreted in the urine (Figure 6) (44). Consistent with other studies, this study found the *N*-acetylcysteine conjugate as the primary metabolite in the urine samples (44, 48). However, compared to studies that examined timed urine samples over a longer period of time (8 h and longer), we found lower ratios of SFN-cysteine/total metabolites and no detectable levels of SFN-glutathione and SFN-cysteine-glycine (44, 48). We hypothesize that the longer period of observation and timed collection of urine samples could explain the differences in ratios. The results of Medina et al. (51), which showed that the SFN-cysteine/total metabolites ratio was twice as high in the first 12 h as in the subsequent 12 h, are in line with this hypothesis. Future research into the relationship between different metabolite ratios and time differences could provide an explanation for these inconsistencies.

Polymorphisms in phase II enzymes involved in the mercapturic acid pathway may help explain the individual outcomes of sulforaphane interventions (Table 1; Figure 6) (58, 59). In the current study, we found that not the enzymes directly involved, but an antioxidant enzyme, NQO1, indirectly involved in this metabolic route influenced sulforaphane secretion. Previous studies showed that the null NQO1*2 polymorphism results in a lack of functional NQO1 protein due to reduced stability, the ability to bind flavin adenine dinucleotide, and a dramatically reduced half-life due to rapid polyubiquitination and proteasomal degradation (83, 90). The lack of functional NQO1 would then in turn reduce the pool of other enzymes that catalyze detoxification of electrophiles, e.g., GSTs (91, 92). We hypothesize that because sulforaphane is metabolized by GSTs, the reduced glutathione pool will result in longer half-lives of circulating sulforaphane and potentially greater systemic effects of cruciferous vegetables. Paradoxically, the increased activation of Nrf2/ARE-dependent genes by sulforaphane, including GSTs, thioredoxin and heme oxygenase 1, would also result in more non-functional NQO1 in individuals with the NQO1*2 polymorphism (41). This positive feedback loop could explain the lower excretion of sulforaphane in participants carrying the polymorphic variant NQO1*2, while no initial differences in antithrombotic effect were observed after a single administration of broccoli sprouts. Repeated-dose studies could reveal whether individuals with genetic polymorphisms that reduce the activity of detoxification enzymes could benefit even more from consumption of cruciferous vegetables. Further clarification of the interactions between polymorphisms and the downstream effectors of sulforaphane are promising new lines of research to elucidate the relationship between the consumption of cruciferous vegetables and human health and well-being (58).

### 4.4. Food-derived sulforaphane has implications for functional food innovation

Overall, only a limited number of food-derived compounds have been clinically investigated for their antiplatelet effects. Some success has been achieved in developing antiplatelet nutraceuticals, with WSTC to date being the only functional food proven to function as a natural cardio-protective functional ingredient, as assessed by the EFSA (25). Therefore, Fruitflow^®^, the trademarked name of WSTC, is authorized by the European Commission to use the health claim (new function claims, Art. 13.5): “water-soluble tomato concentrate (WSTC) I and II helps maintain normal platelet aggregation, which contributes to healthy blood flow” (93, 94). To provide stakeholders with greater clarity on which effects related to cardiovascular health could be studied to support health claims, in 2018, a guidance was published by EFSA’s Panel on Dietetic Products, Nutrition and Allergies (NDA Panel). This guidance document provides more detailed guidelines for the evaluation of Articles 13.1, 13.5, and 14 health claims in this area (95). According to the Panel, a reduction in platelet aggregation [i.e., the percentage of inhibition of platelet aggregation using light transmission aggregometry (LTA) according to well-accepted and standardized protocols] in subjects with platelet activation during sustained exposure to the food/constituent (at least 4 weeks) is a beneficial physiological effect. Other outcome variables, such as thromboxane A_2_ (TXA_2_) or plasma soluble P-selectin, are not considered established markers of platelet aggregation, but can be used as supporting evidence for the scientific substantiation of these claims (95, 96).

Although LTA has been considered the gold standard to assess platelet function for over 40 years, poor standardization and the required manipulation by a skilled technician limit its use to specialized laboratories (20, 97–100). Numerous platelet function tests are currently available; however, their methodologies are diverse, and little is known about the comparability or interchangeability of these tests (20, 97). O’Kennedy et al. examined TXA_2_ levels (via thromboxane B_2_, the stable metabolite of TXA_2_) after a single dose of Fruitflow^®^ (65 mg tomato total active fraction), or 75 mg aspirin (ASA) and found a reduction of approximately 25% at 3 h and 37% at 5 h for WSTC and 66% for ASA after 5 h (26, 94). Our results indicate that sulforaphane seems more effective in reducing overall platelet aggregation at lower doses. In addition, the correlations between sulforaphane and thromboxane demonstrate causation, which is the last criterion in the evaluation of a scientific health claim dossier by EFSA (51, 101, 102). A logical next step for future research would therefore be to investigate the effects of sulforaphane on inhibition of platelet aggregation using LTA in subjects with platelet activation.

### 4.5. Limitations and future directions

This study is the first to demonstrate that urinary 11-dehydro-TXB_2_ is a sensitive, easy-to-use, and suitable biomarker to investigate the effects of phytonutrients on platelet aggregation in young healthy participants who are metabolically challenged in a short period of time. This study is not without limitations. First, the glucoraphanin content of the active sprout material and placebo may have differed from product labels and may have been adversely affected by cultivation conditions, batch-to-batch variation, and preparation methods. Second, it is challenging to find an impeccable placebo in nutritional intervention studies, especially for whole foods in a double-blinded setting. In current study, the placebo had to match the broccoli sprouts in terms of nutrient value, except for sulforaphane content. Despite our efforts, the increased amount of β-carotene and some vitamins in the pea sprouts may have counteracted the expected post-challenge increase in 11-dehydro-TXB_2_ (79, 80). In addition, follow-up studies with a larger sample size will shed more light on the effects of interindividual genetic variability with respect to cruciferous vegetable consumption. Nevertheless, this study has increased our understanding of the antithrombotic effects of sulforaphane and can be used as supporting evidence for the scientific substantiation of claims on the reduction of platelet aggregation (95, 96). When follow-up studies on the effects of sulforaphane using LTA yield positive results, fresh broccoli sprouts or other produce containing enough sulforaphane per serving, could apply for the same authorized claim as WSTC.

## 5. Conclusion

This study demonstrated that a single administration of broccoli sprouts reduced urinary 11-dehydro-TXB_2_ levels by clinically relevant amounts in healthy participants exposed to a standardized caloric load. In addition, the correlations between sulforaphane and thromboxane indicate a causal relationship, which is not influenced by the co-ingestion of the metabolic challenge. 11-Dehydro-TXB_2_ shows promise as a non-invasive, sensitive, and suitable biomarker to investigate the acute effects of phytonutrients on platelet aggregation within hours. Genetic predisposition may influence the health effects of cruciferous vegetable consumption, but more research is needed to ultimately provide personalized dietary advice for consumers. This study forms the basis for a scientific substantiation of claims on the reduction of platelet aggregation for fresh produce containing sulforaphane in the future.

## Data availability statement

The data presented in the study are deposited in the European Variation Archive (EVA) at EMBL-EBI repository under accession number PRJEB63376.

## Ethics statement

The study protocol (NL77272.068.21) was approved by the Medical Ethics Review Committee of Maastricht University Medical Centre+ (MUMC+) and Maastricht University, Maastricht, the Netherlands. The patients/participants provided their written informed consent to participate in this study.

## Author contributions

HS: conceptualization, investigation, formal analysis, visualization, and writing—original draft. EW, EK, MB, and JD: investigation and formal analysis. JB: investigation, formal analysis, and writing—review and editing. TL and FT: writing—review and editing. FO: methodology and writing—review and editing. ABa: conceptualization, writing—review and editing, and supervision. KS: conceptualization, data curation, methodology, visualization, and writing—review and editing. ABo: conceptualization, writing—review and editing, funding acquisition, and supervision. All authors contributed to the article and approved the submitted version.

## Funding

This research was funded by the Dutch Topsector Tuinbouw & Uitgangsmaterialen, grant number TU1118.

## Conflict of interest

MB and JD were employed by Omnigen B.V.

The remaining authors declare that the research was conducted in the absence of any commercial or financial relationships that could be construed as a potential conflict of interest.

## Publisher’s note

All claims expressed in this article are solely those of the authors and do not necessarily represent those of their affiliated organizations, or those of the publisher, the editors and the reviewers. Any product that may be evaluated in this article, or claim that may be made by its manufacturer, is not guaranteed or endorsed by the publisher.
