# Supplementary material for: The beneficial effect of sulforaphane on platelet responsiveness during caloric load: a single-intake, double-blind, placebo-controlled, crossover trial in healthy participants
Source: Front Nutr. 2023 Jul 6;10:1204561. doi: 10.3389/fnut.2023.1204561 (PMC10359317; doi:10.3389/fnut.2023.1204561)
Supplement: Supplementary file 1 [file Table_1.docx]

**Supplementary Table 1. Stargazer results for NAT1.**

| **Participant number** | **Haplotype 1 main * allele** | **Haplotype 2 main * allele** | **Haplotype 1 candidate * alleles** | **Haplotype 2 candidate * alleles** | **Haplotype 1 activity score** | **Haplotype 2 activity score** | **Combined activity score** | **Phenotype** |
| --- | --- | --- | --- | --- | --- | --- | --- | --- |
| 230 | *1 | *1 | *1 | *1 | 1 | 1 | 2 | normal metabolizer |
| 204 | *1 | *1 | *1 | *1 | 1 | 1 | 2 | normal metabolizer |
| 303 | *1 | *11 | *1 | *11, *30 | 1 | unknown | unknown | unknown^#^ |
| 454 | *1 | *1 | *1 | *1 | 1 | 1 | 2 | normal metabolizer |
| 422 | *1 | *1 | *1 | *1 | 1 | 1 | 2 | normal metabolizer |
| 919 | *1 | *1 | *1 | *1 | 1 | 1 | 2 | normal metabolizer |
| 084 | *1 | *1 | *1 | *1 | 1 | 1 | 2 | normal metabolizer |
| 740 | *1 | *1 | *1 | *1 | 1 | 1 | 2 | normal metabolizer |
| 323 | *1 | *14 | *1 | *14 | 1 | 0.5 | 1.5 | normal metabolizer |
| 771 | *1 | *1 | *1 | *1 | 1 | 1 | 2 | normal metabolizer |
| 178 | *1 | *1 | *1 | *1 | 1 | 1 | 2 | normal metabolizer |
| 029 | *1 | *1 | *1 | *1 | 1 | 1 | 2 | normal metabolizer |

*Note: * alleles are determined for both haplotypes and corresponding activity scores are added. The combined activity score translates to the metabolizer phenotype where poor metabolizers have a combined activity score of 0, intermediate metabolizers between 0 and 1 normal metabolizers between 1 and 2, rapid metabolizers between 2 and 2.5 and ultrarapid metabolizers larger than 2.5.* *^#^ Stargazer phenotyped all but one participant as normal metabolizers by the pharmacogene NAT1 and was unable to determine the metabolism phenotype for one participant due to both NAT1*11 and NAT1*30 having an unknown activity score.*

**Supplementary Table 2. Stargazer results for NAT2.**

| **Participant number** | **Haplotype 1 main * allele** | **Haplotype 2 main * allele** | **Haplotype 1 candidate * alleles** | **Haplotype 2 candidate * alleles** | **Haplotype 1 activity score** | **Haplotype 2 activity score** | **Combined activity score** | **Phenotype** |
| --- | --- | --- | --- | --- | --- | --- | --- | --- |
| 230 | *1 | *1 | *1 | *1 | 1 | 1 | 2 | normal metabolizer |
| 204 | *1 | *6 | *1 | *6, *13 | 1 | 0.5 | 1.5 | normal metabolizer |
| 303 | *1 | *6 | *1 | *6, *13 | 1 | 0.5 | 1.5 | normal metabolizer |
| 454 | *1 | *6 | *1 | *6, *13 | 1 | 0.5 | 1.5 | normal metabolizer |
| 422 | *1 | *6 | *1 | *6, *13 | 1 | 0.5 | 1.5 | normal metabolizer |
| 919 | *1 | *1 | *1 | *1 | 1 | 1 | 2 | normal metabolizer |
| 084 | *1 | *1 | *1 | *1 | 1 | 1 | 2 | normal metabolizer |
| 740 | *5 | *6 | *5, *11 | *6, *13 | 0.5 | 0.5 | 1 | intermediate metabolizer |
| 323 | *1 | *5 | *1 | *5, *11 | 1 | 0.5 | 1.5 | normal metabolizer |
| 771 | *1 | *5 | *1 | *5, *11 | 1 | 0.5 | 1.5 | normal metabolizer |
| 178 | *5 | *6 | *5, *11 | *6, *13 | 0.5 | 0.5 | 1 | intermediate metabolizer |
| 029 | *6 | *6 | *6, *13 | *6, *13 | 0.5 | 0.5 | 1 | intermediate metabolizer |

*Note: * alleles are determined for both haplotypes and corresponding activity scores are added. The combined activity score translates to the metabolizer phenotype where poor metabolizers have a combined activity score of 0, intermediate metabolizers between 0 and 1 normal metabolizers between 1 and 2, rapid metabolizers between 2 and 2.5 and ultrarapid metabolizers larger than 2.5.*
